# Breath restored: yoga-based training lowers estimated lung age among individuals with opium dependence in a residential pre-post study

**DOI:** 10.3389/fpsyt.2026.1759745

**Published:** 2026-02-24

**Authors:** Arjun Ram Roj, Surajnath Siddh, Harish Sharma, Dhananjay Foujdar, Ekta Mishra, Sonu Kumar, K.M. Vartika, Megha Pundir, Gautam Mishra, Sarashti Saini, Sanjib Patra

**Affiliations:** 1 Department of Yoga, Central University of Rajasthan, Ajmer, India; 2 Department of Yoga, Kalinga University, Naya Raipur, India; 3 Integrative Medicine, National Institute of Mental Health and Neurosciences, Bengaluru, India

**Keywords:** addiction, addiction management, complemenfary and alternative medicine, opioid induced respiratory depression, opioid use disorder (OUD), opium addiction, pranayama, yoga

## Abstract

**Background:**

Chronic opium use is associated with respiratory impairment and increased risk of respiratory-related mortality. We evaluated whether a structured, breath-centred *yoga* programme could improve pulmonary function in men with opium dependence.

**Methods:**

In this single-arm pre–post feasibility study, 38 men were enrolled during a 1-month residential de-addiction programme and 30 completers (mean age 43.5 ± 12.2 years) underwent spirometry at baseline and after a month of twice-daily, instructor-led breath-based *yoga* (*pranayama*, *asana*-linked breathing, and relaxation). Primary outcomes were within-group changes in forced vital capacity (FVC), forced expiratory volume in 1 s (FEV_1_), FEV_1_/FVC, peak expiratory flow rate (PEFR), mid-expiratory flows, and estimated lung age; paired t-tests and Cohen’s d were used for inference.

**Results:**

The intervention was associated with statistically significant increases in FVC (2.76 ± 0.94 to 3.37 ± 0.73 L; mean change +0.61 L; p < 0.001; d = 0.88) and PEFR (4.45 ± 1.95 to 6.09 ± 1.97 L·s^−^¹; +1.64; p < 0.001; d = 0.84), and a moderate increase in FEV_1_ (2.34 ± 0.88 to 2.71 ± 0.79 L; +0.37; p = 0.011; d = 0.49). FEV_1_/FVC did not change significantly (85.65 ± 9.41% to 82.15 ± 17.55%; p = 0.404). Estimated lung age decreased by a mean of 6.72 years (47.96 ± 16.87 to 41.24 ± 14.00 years; p = 0.003; d = 0.59). No serious adverse events were reported.

**Conclusions:**

A month of structured breath-based *yoga* produced clinically and statistically meaningful improvements in lung volumes, expiratory flow, and estimated lung age in men with chronic opium dependence. These pilot data support testing this culturally acceptable, low-cost intervention in larger, randomized trials to confirm efficacy, explore mechanisms, and determine durability.

**Trial registration:**

CTRI/2025/07/089999

## 1 Introduction

Substance use disorders constitute a growing global health concern, contributing substantially to premature mortality, morbidity, and socioeconomic burden (1). Drug use results in 0.6 million fatalities, 80% of which are from opioids. Opioids represent the most lethal, with an estimated 125, 000 opioid-related deaths occurring worldwide in 2019, primarily due to overdose and associated complications (2). The mechanism underlying this elevated mortality risk is predominantly opioid-induced respiratory depression (OIRD), which alone accounts for nearly 80, 000 of opioid-related deaths (3). Chronic opioid use can lead to secondary ventilatory impairments beyond central respiratory depression, such as increased respiratory muscle rigidity and decreased chest wall compliance that constrain ventilation and reduce tidal volume, further impairing ventilatory mechanics (4, 5). Despite advances in medical management, the persistence of this complication underscores the urgent need for innovative and adjunctive strategies to address opioid induced respiratory complications, reducing opioid-related mortality.

In India, opioids constitute one of the most widely misused substances, with prevalence rates surpassing the global average (6). Among these, opium, a natural plant-derived substance from which morphine and other opioids are synthesized, carries particular historical and cultural significance in the state of Rajasthan. Opium consumption has long been interwoven with social, religious, and community practices, especially in the western regions of the state (7). This epidemic is concentrated in some regions: in rural western Rajasthan crude opium use is socially sanctioned and exceptionally common – epidemiological studies report that roughly 8–13% of adult men use opium in that area, a rate that has been described as the world’s highest opium addiction prevalence (8). These figures underscore the urgent need to address opium dependence in India and especially in Rajasthan. This cultural acceptance not only normalizes use but also perpetuates misconceptions, the most prominent being the belief that discontinuing opium results in early death. Such beliefs act as barriers to de-addiction efforts and reinforce dependency beyond the pharmacological effects of the drug (9).

Biomedical realities inadvertently lend weight to this cultural notion. The majority of existing de-addiction modalities, including opioid substitution therapy (OST), primarily focus on reducing withdrawal distress and controlling craving through modulation of the brain’s reward circuitry (10). However, OST and other pharmacological treatments not only fail to address OIRD and its secondary ventilatory deficits, but in certain contexts may even contribute to respiratory suppression (11). This disconnect has serious implications: the community perception that cessation of opium leads to death aligns with the untreated risk of respiratory compromise. Consequently, treatment adherence remains low, relapse rates high, and only a small fraction of affected individuals actively seek care, perpetuating the cycle of opium dependence in this region.

These challenges highlight the urgent need for non-pharmacological adjuncts that can target the secondary ventilatory deficits of OIRD while being culturally acceptable and feasible. *Yoga*, a traditional Indian practice with growing global recognition, offers promising potential in this context (12). Prior research has demonstrated the role of *yoga*-based practices, especially breath regulation techniques (*pranayama*), in enhancing pulmonary function, improving autonomic balance, and strengthening respiratory musculature (13). Additionally, *yoga* interventions have shown efficacy as supportive strategies in substance abuse rehabilitation, promoting self-regulation and resilience (12). These findings suggest that *yoga* may serve a dual role: directly alleviating respiratory risks associated with opioid use and simultaneously increasing community acceptance of de-addiction programs by aligning with indigenous traditions.

However, despite this potential, the specific application of *yoga* as an intervention for secondary ventilatory deficits of OIRD remains largely unexplored. No studies to date have systematically evaluated the effects of a structured *yoga* protocol on pulmonary function in individuals with chronic opium use. Bridging this gap is of both scientific and clinical importance. By addressing the secondary ventilatory deficits of OIRD, primary cause of opioid-related mortality through a low-cost, culturally resonant intervention, such research can lay the foundation for improving treatment adherence, reducing relapse rates, and ultimately lowering the burden of opioid-related deaths in high-risk populations such as those in Western Rajasthan, India.

## 2 Materials and methods

### 2.1 Study design

This study represents Phase 1 (single-group pre-post feasibility phase) of a registered research program (CTRI/2025/07/089999). Phase 1 and Phase 2 (planned RCT) share identical outcome measures, assessment protocols, and target population, ensuring that Phase 1 results directly inform Phase 2 design parameters and sample size estimations. This was a single-arm, pre–post interventional study evaluating the effect of a structured breath-based *yoga* program on pulmonary function in individuals with opium dependence. The study received approval from the Institutional Human Ethics Committee at the Central University of Rajasthan prior to participant recruitment and data collection. After confirming eligibility, baseline data including demographics and spirometry assessment was done, the study was conducted during a residential de-addiction camp organized at the Jasnath Asan premises in Khimsar, Rajasthan. The intervention lasted 1 month and spirometry was performed at baseline (day 0) and post-intervention (day 30).

### 2.2 Sample size

Sample size was determined to detect a pre–post change in the FEV_1_/FVC ratio following a *pranayama*-based breathing intervention, using an effect size of d = 0.725 derived from a previously published *pranayama* study, calculated through the G*Power software (version 3.1) (14, 15). Statistical power analysis was performed employing a two-tailed one-sample t-test framework, assuming a significance level of α = 0.05 and a desired statistical power of 95%. Based on the effect size parameters and standard deviation estimates obtained from the prior *pranayama* literature, a minimum total sample of 27 participants was required to achieve the targeted power. Accounting for an anticipated attrition rate of 30%, the final planned sample size was increased to 38 participants to maintain sufficient statistical precision.

### 2.3 Participants

#### 2.3.1 Recruitment

**Figure 1:**
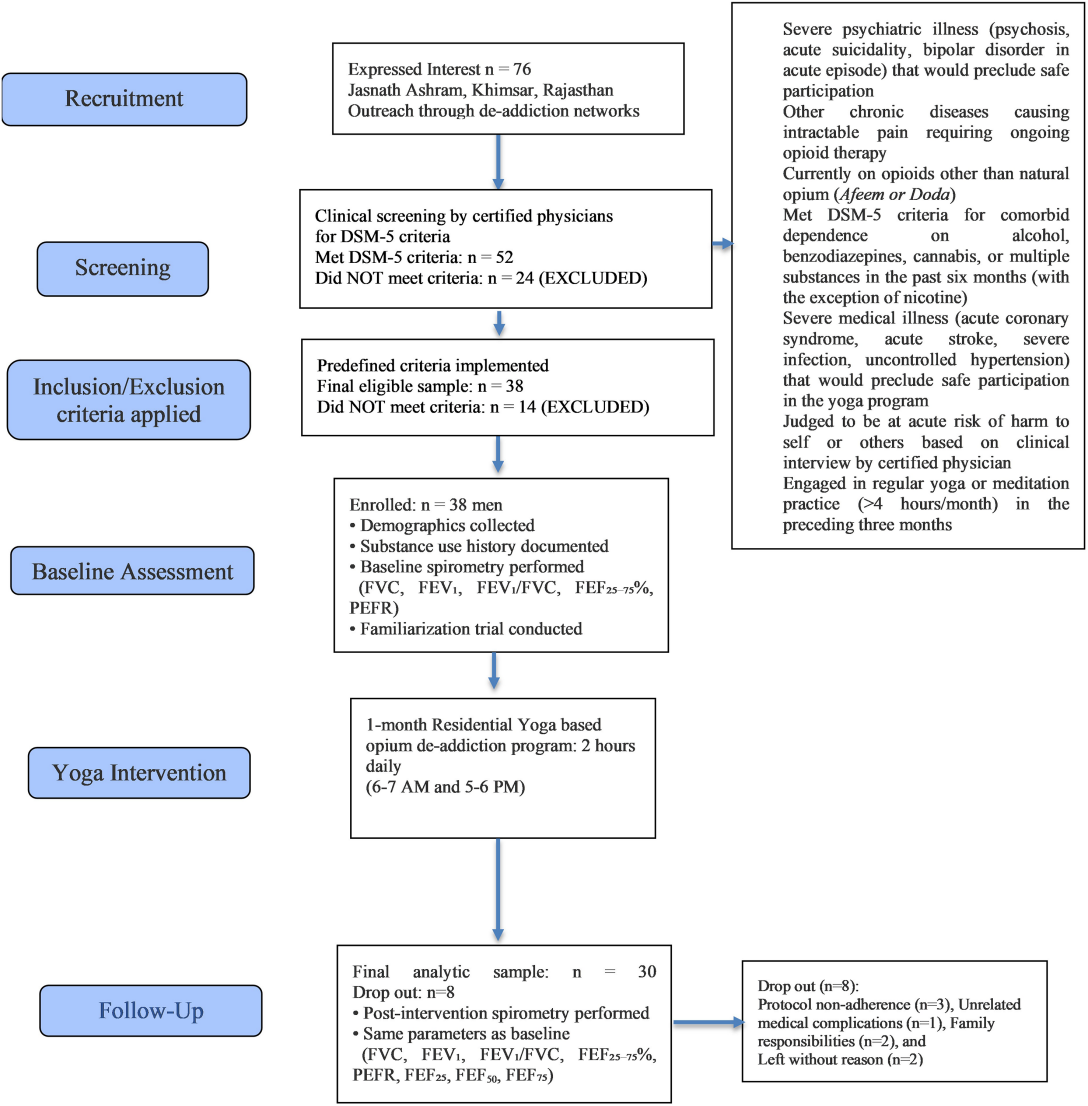
Consort flowchart.

Participants were recruited through clinical screening conducted during a residential de-addiction program organized at the Jasnath Asan in Khimsar, Rajasthan, India. Recruitment involved direct outreach through local de-addiction networks, community health workers, and treatment referral centers in Western Rajasthan. Total 76 individuals expressed their interest in the residential rehabilitation program and were invited to participate in the baseline screening at the facility. Information about the study, including inclusion and exclusion criteria and the program requirements, was provided verbally and in written form. All prospective participants provided written informed consent prior to undergoing screening procedures (see **Figure 1**).

#### 2.3.2 Screening

Eligible candidates underwent structured clinical screening performed by certified addiction medicine physicians. Screening included a comprehensive clinical interview assessing psychiatric status, medical history, substance use patterns, and current medication use. All participants were assessed for DSM-5 criteria for opioid use disorder (opium dependence) using the Structured Clinical Interview for DSM-5 (SCID-5) (16). Total 52 participants met DSM-5 criteria for opioid use disorder and those who didn’t (n=24) were excluded and referred for appropriate clinical management (see **Figure 1**).

#### 2.3.3 Inclusion and exclusion criteria

Participants were eligible for inclusion if they were male and between 18 and 60 years of age, met the DSM-5 diagnostic criteria for opioid use disorder (opium dependence) as established through the Structured Clinical Interview for DSM-5 (SCID-5), were permanent residents of Western Rajasthan, and were both willing and able to participate fully in the 1-month residential rehabilitation program. Provision of written informed consent was mandatory for enrolment.

Individuals were excluded if they were diagnosed with severe psychiatric illnesses such as psychosis, acute suicidality, or bipolar disorder in an acute episode that could compromise safe participation; if they had chronic medical conditions causing intractable pain necessitating ongoing opioid therapy; or if they were currently using opioids other than natural opium (*Afeem or Doda*). Additional exclusion criteria included fulfilment of DSM-5 criteria for comorbid dependence on alcohol, benzodiazepines, cannabis, or multiple substances (except nicotine) within the previous six months; the presence of severe medical conditions including acute coronary syndrome, acute stroke, severe infection, or uncontrolled hypertension that would preclude safe participation in the *yoga* program; being judged at acute risk of harm to self or others based on a clinical interview by a certified physician; or having engaged in regular *yoga* or meditation practice exceeding four hours per month in the preceding three months.

#### 2.3.4 Demographic characteristics

Of the initial candidate pool, 14 participants failed to satisfy the study’s eligibility requirements and were excluded. Comprehensive baseline data were obtained from the 38 eligible participants, including demographic variables (age, marital status, residence, education, employment), substance use characteristics (daily dose in grams, duration of use in years, DSM-5 dependence severity score), family addiction history, and personal monthly income (see **Figure 1**, **Table 1**).

**Table 1:** Demographic details.

Variable	Mean ± SD or n (%)
Age (years)	43.5 ± 12.2
Married	30 (100%)
Living with family	30 (100%)
Vegetarian	30 (100%)
Occupation	
Driver	4 (13%)
Farmer	19 (63%)
Other	7 (23%)
Education level	
Uneducated	8 (26%)
5th Standard	6 (20%)
8th Standard	7 (23%)
10th Standard	5 (16%)
Higher studies	4 (13%)
Annual net savings (after household expenses)	
<₹40, 000	26 (86%)
₹40, 000–₹100, 000	2 (6%)
>₹100, 000	2 (6%)
Addiction	
Addiction in Family	10 (33%)
DSM-5 Opioid Use Disorder Criteria Met	7.87 ± 1.36
Duration of Opioid Use (years)	17.03 ± 8.30
Average Daily Dose (grams)	2.29 ± 1.34

### 2.4 Outcome measures

#### 2.4.1 Spirometry testing

Lung function testing was performed using a portable spirometer (RMS Helios 401, Chandigarh, India) (17, 18). Daily equipment verification was performed with a 3-liter calibration syringe. spirometry testing adhered to ATS/ERS technical standards for acceptability and reproducibility (19). Participants were tested at the same time of day, after ≥12 h opium abstinence, in a seated position wearing a nose clip and completed at least three acceptable, reproducible expiratory maneuvers, and the highest values were considered for analysis. A trained technician supervised all assessments to ensure procedure quality. Spirometry variables measured included FVC, FEV_1_, FEV_1_/FVC ratio, FEF_25–75_%, and PEFR. A familiarization trial was provided to minimize learning effects.

### 2.5 Safety and adverse events

Breathing based *yoga* practices are regarded as safe, minimally invasive therapeutic interventions with a low adverse event profile when administered according to validated, standardized protocol (20). Recent systematic literature review and quantitative synthesis of existing evidence demonstrated that breathing-centred *yoga* practices yielded substantial gains in aerobic exercise capacity and measured pulmonary function parameters. Similar breathing based *yogic* practices has been used in addiction studies, with no report of adverse events (12, 21). The breathing-based intervention module was designed by qualified *yoga* therapy specialist.

### 2.6 Intervention

Intervention was a residential-based programme delivered by a certified *yoga* therapist, consisting of two daily sessions of 1 hour each (6:00–7:00 a.m. and 5:00–6:00 p.m. IST), conducted over a period of 1 month. It consists of breath-based *yoga* module comprising of practice sessions. The intervention included a set of breathing-based exercises, *Suryanamaskara, Pranayamas* and was concluded with relaxation techniques. The structure of this intervention program is listed below in the (See **Table 2**).

**Table 2:** Structure and components of the four-week breath-based yoga intervention delivered during residential treatment.

Phase	Practice (*Sanskrit*/English)	Time (rounds/cycles per round/rest between round/rest post practice)	Specific technique	Physiological & biomechanical target
I. Centering	Prayer 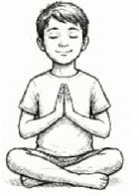	1 min	Chanting with eyes closed	Psychophysiological Grounding
II. Preparatory	*Kapalbhati* (Frontal Brain Cleansing) 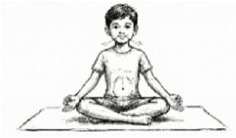	3 mins (3 Rounds; 30 strokes/round; 30 secs active + 30 secs rest/retention per round)	Active forceful exhalation, passive inhalation	Expiratory Muscle Conditioning: High-intensity isotonic training for abdominal and internal intercostal muscles; clears airway secretions.
III. Thoracic Mobilization	*Trikonasana* Variation (Side Bending) 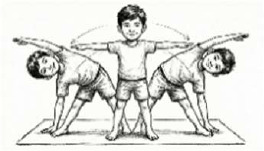	2 mins (1 Round; 5 cycles; Lateral flexion; 1.5 mins practice + 30 secs rest)	Lateral flexion with synchronized breathing	Lateral Chest Expansion: Stretches intercostal muscles; opens lateral lung zones to improve compliance.
	Hands In-and-Out Breathing 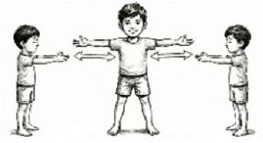	1.5 mins (1 Round; 5 Cycles; 1 min practice + 30 secs rest)	Dynamic arm spread with deep inspiration	"Bucket-Handle" Motion: Maximizes transverse diameter of the thorax; stretches pectoralis major to reduce kyphotic rigidity.
	Hand Stretch Breathing 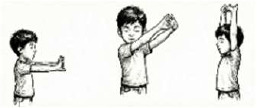	1.5 mins (1 Round; 3 Cycles; 1 min practice + 30 secs rest)	Arms 90°/135°/180° stretch	"Pump-Handle" Motion: Mobilizes costovertebral joints; expands anterior-posterior thoracic diameter.
	Ankle Stretch Breathing 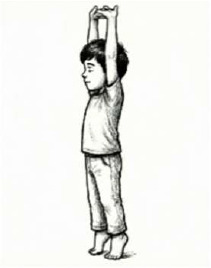	1 min (1 Round; 3 Cycles; 45 secs practice + 15 secs rest)	Heel raise with vertical arm stretch	Vertical Thoracic Expansion: Elongates the thoracic cavity; stimulates venous return (calf muscle pump).
IV. Asana-Based Breathing	*Shashankasana* Breathing (Rabbit Pose) 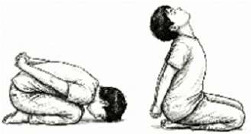	2.5 mins (2 Rounds; 5 Cycles; 1.5 mins active + 30 sec rest)	Forward flexion with abdominal compression	Posterior Basal Expansion: Restricts anterior abdominal excursion, forcing diaphragmatic movement posteriorly to ventilate dependent lung zones.
	*Bhujangasana* Breathing (Cobra Pose) 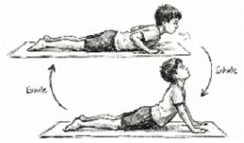	2.5 mins (2 Rounds; 5 Cycles; 1.5 min active + 1 min rest)	Prone extension with deep inhalation	Anterior Apical Recruitment: Increases intra-abdominal pressure, mechanically expanding the anterior chest wall; counteracts "slouched" posture.
	*Setu Bandhasana* Breathing (Bridge Pose) 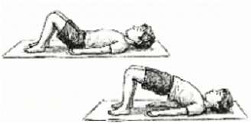	2.5 mins (2 Rounds; 5 Cycles; 1.5 min active + 1 min rest)	Supine pelvic lift	Diaphragmatic Strengthening: Forces diaphragm to contract against the gravity of abdominal viscera; recruits apical lung segments.
	*Pavanamuktasana* Breathing (Wind-Relieving Pose) 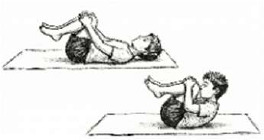	2.5 mins (2 Rounds; 5 Cycles; 1.5 min active + 1 min rest)	Thigh-to-chest compression	Intra-abdominal Pressure Regulation: Massages abdominal viscera; improves diaphragm fluidity by alternating compression and release.
V. Neuromuscular Reset	QRT (Quick Relaxation Technique) 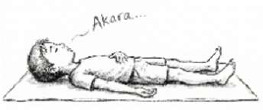	5 Mins	Guided body scan (supine)	Autonomic Switch: Rapid reduction of muscle tone; facilitates shift from sympathetic to parasympathetic dominance.
VI. Dynamic Activation	*Surya Namaskara* (Sun Salutation) 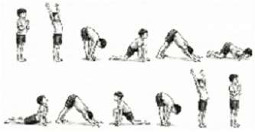	5 mins (2 Rounds; 2 Rapid Cycles; 4 min active + 1 min rest)	12-step dynamic sequence	Cardiorespiratory Endurance: Increases cardiac output and minute ventilation; integrates breath with gross motor movement.
VII. Deep Relaxation	DRT (Deep Relaxation Technique) 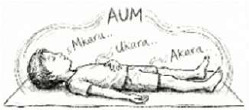	10 Mins	Supine, progressive relaxation	Metabolic Downregulation: Lowers oxygen consumption (VO2 max); reduces residual neuromuscular tension.
VIII. Pranayama	Sectional Breathing (*Vibhagiya Pranayama*) 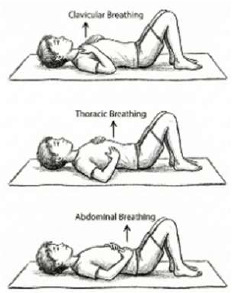	6.5 mins (5 Rounds; 6 Cycles; 5 mins active; 1.5 mins rest)	Abdominal, Thoracic, Clavicular isolation	Ventilation Distribution: Re-trains the "wave" of breathing; specifically recruits under-ventilated lung segments (apical/basal).
	*Ujjayi* Breathing (Victorious Breath) 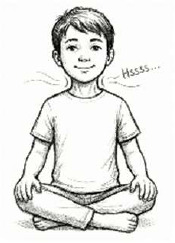	6 mins (3 Rounds; 10 Cycles; 4.5 mins active; 1.5 mins rest)	Glottal constriction with friction sound	Positive End-Expiratory Pressure (PEEP): Increases intrabronchial pressure to prevent distal airway collapse; enhances vagal tone.
	*Bhramari* (Humming Bee Breath) 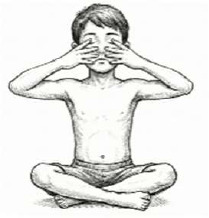	6 mins (4 Rounds; 6 Cycles; 4 min active; 2 min rest)	Prolonged exhalation with humming	Biochemical Modulation: Oscillatory sound vibration increases nasal Nitric Oxide (NO) production (vasodilator/bronchodilator).
IX. Integration	Prayer/Silence 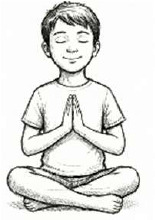	2 mins	Chanting with eyes closed	Final Integrative Settling

The module outlines session frequency, duration, and the specific practices included across breathing exercises, postures, and relaxation elements.

### 2.7 Statistical analysis

Analyses were performed JASP (version 0.17.1, 2023) and sample size estimation was performed with G*Power (15). Descriptive statistics was used to summarise the baseline demographic variables and addiction profile. Within-group changes in spirometry parameters (FVC, FEV_1_, FEV_1_/FVC, PEFR, FEF_25–75_%) from baseline to post-intervention were assessed using paired-sample t-tests. Normality of change scores was assessed using the Shapiro–Wilk test. Results are presented as mean change with 95% confidence intervals. Effect sizes were calculated using Cohen’s d. Two-tailed significance was set at p < 0.05. Analyses included 30 completers (participants with both pre- and post-intervention assessments). Missing data from 8 non-completers were not imputed and assumed missing at random.

## 3 Results

### 3.1 Participant characteristics

76 individuals were screened by community outreach, after screening 52 participants went through eligibility criteria, 38 participants initially enrolled, 30 completed the intervention and post-assessment. Eight participants were lost to follow-up [protocol non-adherence (n=3), unrelated medical complications (n=1), family responsibilities (n=2), and left without reason (n=2)].

### 3.2 Pulmonary outcomes

Spirometry assessments were conducted in accordance with ATS/ERS standards for reproducibility and acceptability. The intervention was associated with statistically significant improvements in multiple domains of pulmonary function, including lung volumes, large airway flow rates, and derived indices of lung age. A summary of all pulmonary parameters is provided in **Table 3**.

**Table 3:** Pre- and post-intervention spirometry outcomes following 1-month breath-based *yoga* program in individuals with chronic opium dependence.

Parameter	Pre-intervention (mean ± SD)	Post-intervention (mean ± SD)	Mean difference	P-value (paired t-test)	Effect size (Cohen’s d)
Volume metrics					
FVC (L)	2.76 ± 0.94	3.37 ± 0.73	+0.61	< 0.001***	0.879
FEV_1_ (L)	2.34 ± 0.88	2.71 ± 0.79	+0.37	0.011*	0.493
FEV_1_ / FVC (%)	85.65 ± 9.41	82.15 ± 17.55	-3.50	0.404	0.155
Flow metrics					
PEFR (L·s^−^¹)	4.45 ± 1.95	6.09 ± 1.97	+1.64	< 0.001***	0.839
FEF_25_ (L·s^−^¹)	4.01 ± 2.01	5.48 ± 1.98	+1.47	< 0.001***	0.782
FEF_50_ (L·s^−^¹)	3.11 ± 1.57	3.73 ± 1.33	+0.62	0.015*	0.473
FEF_25–75_ (L·s^−^¹)	2.73 ± 1.29	3.05 ± 1.06	+0.32	0.084	0.327
FEF_75_ (L·s^−^¹)	1.61 ± 0.72	1.48 ± 0.65	-0.13	0.339	0.178
Derived indices					
Lung Age (years)	47.96 ± 16.87	41.24 ± 14.00	-6.72	0.003**	0.587

Data are presented as mean ± standard deviation. FVC, Forced Vital Capacity; FEV_1_, Forced Expiratory Volume in 1 second; PEFR, Peak Expiratory Flow Rate; FEF, Forced Expiratory Flow. p-values calculated via paired t-test. (p < 0.05*; p < 0.01**; p < 0.001***).

#### 3.2.1 Lung volumes and capacities

**Figure 2:**
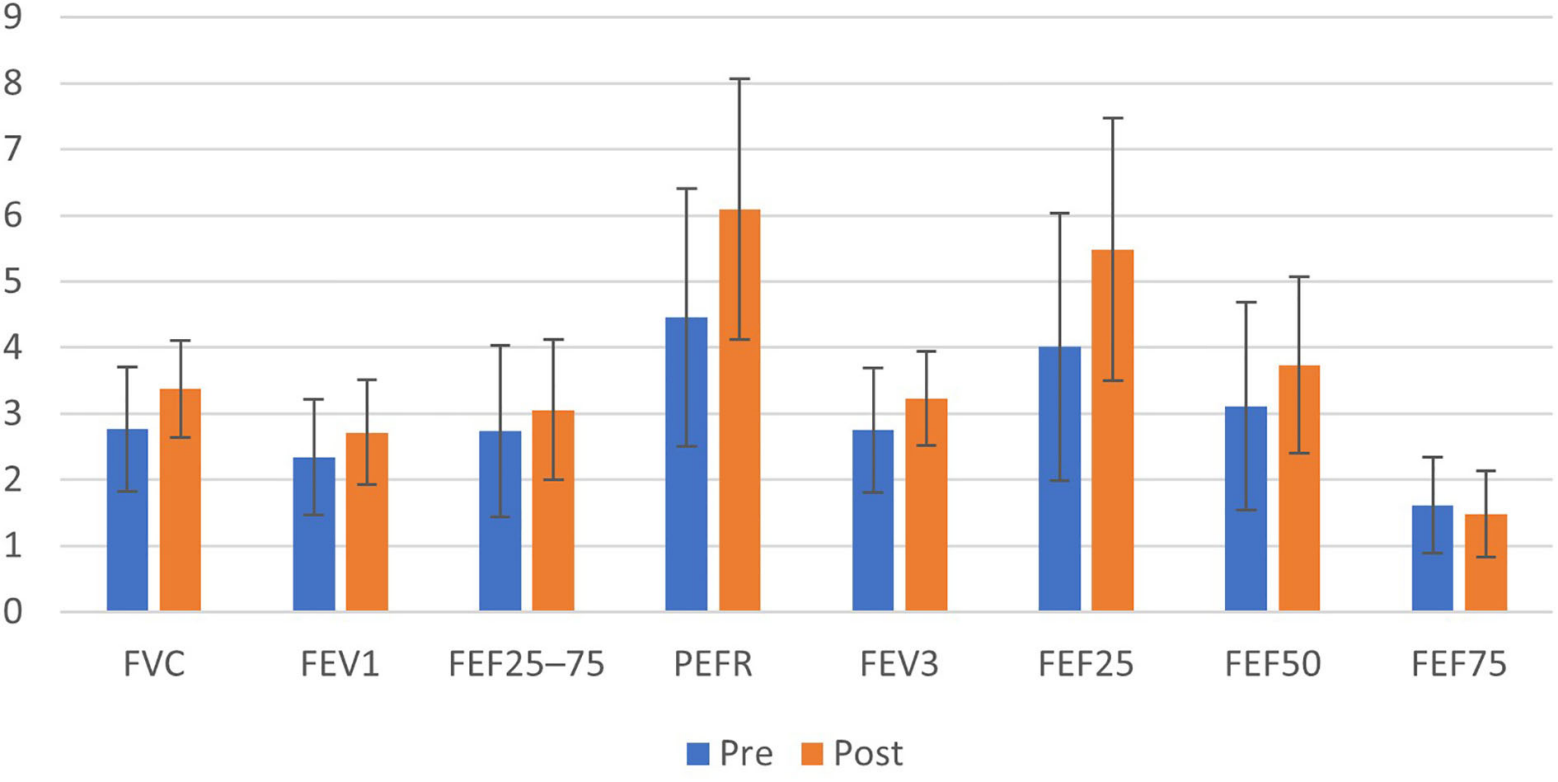
Grouped bar chart showing mean ± SD for spirometry indices (FVC, FEV_1_, FEF25–75, PEFR, FEV3, FEF25, FEF50, FEF75) measured before and after the intervention (n = 30).

A significant increase was observed in Forced Vital Capacity (FVC), which improved from a baseline mean of 2.76 ± 0.94 L to 3.37 ± 0.73 L post-intervention (p < 0.001). This change corresponds to a Cohen’s *d* of 0.879. Similarly, Forced Expiratory Volume in the first second (FEV_1_) increased from 2.34 ± 0.88 L to 2.71 ± 0.79 L (p = 0.011; *d* = 0.493) (See **Figure 2**).

The FEV_1_/FVC ratio, showed a non-significant change from 85.65 ± 9.41% to 82.15 ± 17.55% (*p = 0.404; d=0.155*).

#### 3.2.2 Expiratory flow rate

Peak Expiratory Flow Rate (PEFR), increased significantly from 4.45 ± 1.95 L·s^−^¹ to 6.09 ± 1.97 L·s^−^¹ (*p < 0.001*; *d* = 0.839). FEF_25_ increased from 4.01 ± 2.01 L·s^−^¹ to 5.48 ± 1.98 L·s^−^¹ *(p < 0.001)*, and FEF_50_ increased from 3.11 ± 1.57 L·s^−^¹ to 3.73 ± 1.33 L·s^−^¹ *(p = 0.015)* (See **Figure 2**).

**Figure 3:**
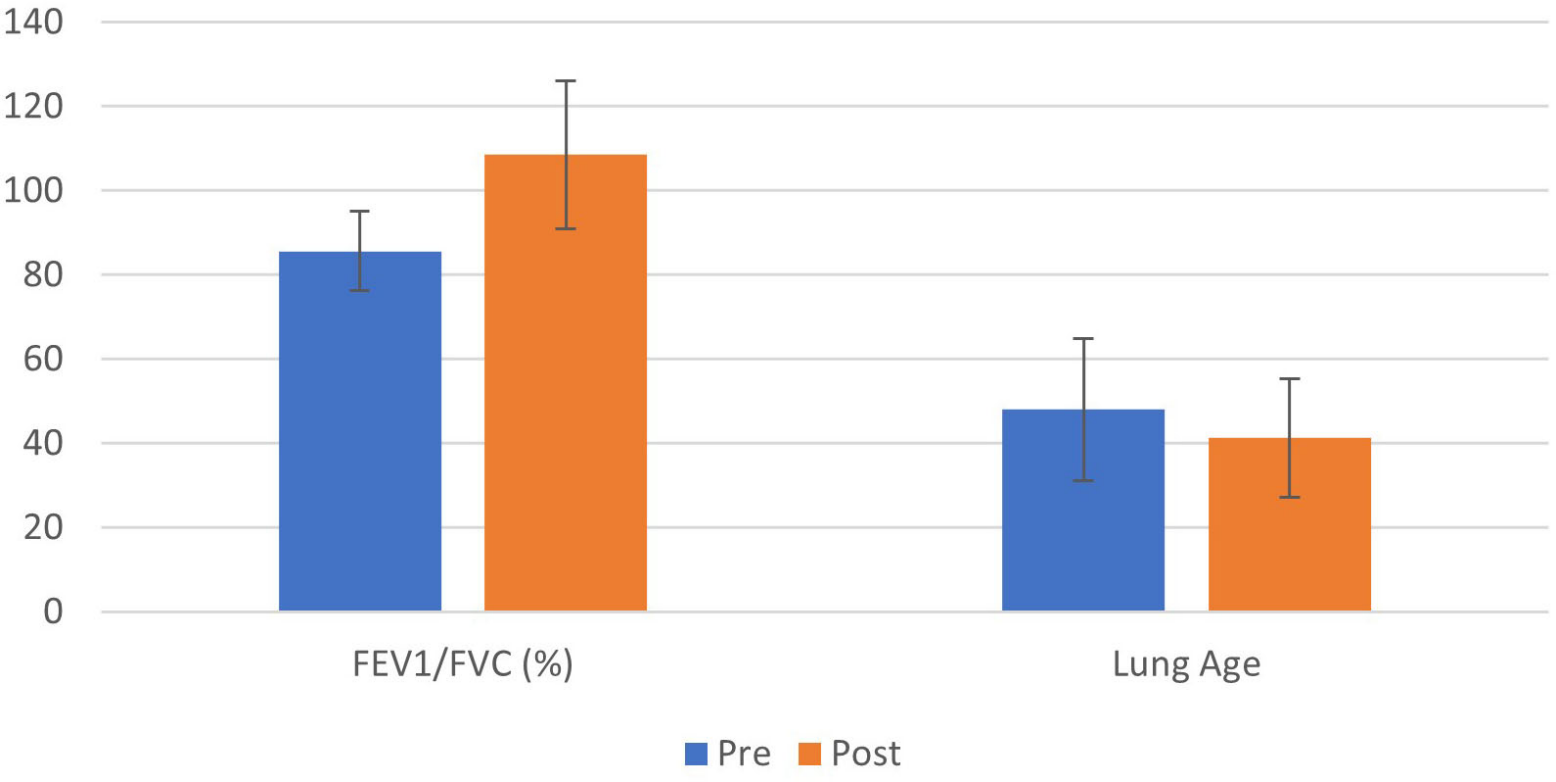
Bar chart shows mean ± SD for FEV_1_/FVC (%) and estimated lung age before and after the breath-based *yoga* intervention (n = 30).

The Forced Expiratory Flow between 25% and 75% of FVC (FEF_25–75_%) showed no statistically significant change (2.73 ± 1.29 L·s^−^¹ vs. 3.05 ± 1.06 L·s^−^¹; *p = 0.084*). Similarly, the expiratory flow (FEF_75_) remained statistically unchanged *(p = 0.339)* (See **Figure 3**).

#### 3.2.3 Lungs age

The estimated Lung Age, derived from spirometry indices, decreased significantly from a pre-intervention mean of 47.96 ± 16.87 years to 41.24 ± 14.00 years *(p = 0.003)*. This represents a mean reduction of approximately 6.7 years in the estimated lung age of the cohort, with a moderate effect size (*d* = 0.587) (See **Figure 3**).

## 4 Discussion

This pre–post study demonstrated that a structured *yoga* intervention significantly improved pulmonary function in opium-dependent men. A significant increase in FVC, FEV_1_, PEFR, FEF_25_, and FEF_50_ were observed following the intervention, while FEV_1_/FVC ratio remained unchanged. These improvements suggest that *yoga* enhances both lung volumes and expiratory flows, reflecting improved respiratory mechanics without altering airway obstruction. Given the chronic respiratory impairment associated with opium use, including bronchitis, fibrosis, and central respiratory depression, these findings carry meaningful clinical relevance.

Results in this study are consistent with existing literature on *yoga’s* impact on lung function. Studies in healthy adults and patients with asthma and COPD have shown similar improvements in FEV_1_, FVC, and PEFR following *yoga* training. A meta-analysis of 15 RCTs in asthma patients reported significant increase in all major spirometry parameters with *yoga*-based interventions (11, 22). In COPD patients, *yoga* has been associated with modest improvements in FEV_1_ and exercise capacity (23).

Existing pharmacological approaches to OIRD focus largely on acute receptor antagonism (naloxone) and modulation of ventilatory drive or opioid exposure such as methadone or buprenorphine maintenance (24–26). Naloxone reliably reverses acute opioid receptor–mediated respiratory arrest but has a short duration of action (25). Similarly, Opioid Substitution Therapies (OST) significantly reduce withdrawal and overdose mortality, though full agonists like methadone, carry their own respiratory risks and do not directly improve baseline respiratory mechanics (26). Other experimental strategies, such as ampakines and potassium-channel modulators, have shown promise in augmenting respiratory drive but remain limited by side-effect profiles and sparse clinical availability (5, 27). Collectively, while these pharmacologic options effectively address receptor blockade or chemical drive, they are not primarily designed to remediate the chronic mechanical deficits (e.g., chest-wall rigidity, diaphragmatic weakness) or autonomic patterning central to persistent restrictive physiology, a functional gap that breath-centred, neuromuscular practices such as *yoga* are plausibly positioned to complement (5).

In order to understand the mechanism of action of the administered *yoga* practices, it was already demonstrated that *yogasanas* (postures) and dynamic stretches probably increase chest wall compliance. Backbends *(Bhujangasana, Setu Bandhasana)*, side-bends, and chest-opening poses would have counteracted the stiffness of the thoracic cage and allowed fuller inhalation. Evidence from a randomized controlled trial of *yoga* therapy in chest trauma patients reported statistically significant improvements in chest wall mobility following *yoga* intervention (28). Similarly, a comparative study of classical breathing exercises and *yoga* on posture and spinal mobility showed that *yoga* significantly improved spinal extension-flexion capacity and corrected postural deviations such as thoracic hyper-kyphosis, restoring thoracic mobility and chest wall compliance that creates the potential for increased lung volumes (29).

In addition, practicing coordinated breathing drills, for example, “sectional breathing” isolating abdominal, thoracic, and clavicular expansion, may have retrained the breathing pattern to be deeper and more efficient. Research on diaphragmatic breathing exercises demonstrated significant improvements in FVC following targeted breathing exercises (30). Further, a clinical trial measuring the effects of proprioceptive neuromuscular facilitation (PNF) respiration pattern exercises found significant improvements in expiratory reserve volume and vital capacity, establishing that neuromuscular retraining through coordinated breathing patterns produces measurable respiratory improvements (31).

Furthermore, consciously coupling breath with limb movements can provide powerful proprioceptive feedback to the respiratory centers, reinforcing the sensation of a full breath. The scientific foundation for this approach derives from established neurophysiology: afferent sensory information from limb muscles directly modulates respiratory motor output through neuronal pathways between lumbar proprioceptive afferents, medullary respiratory networks, and phrenic motoneurons (32–34). However, systematic reviews of proprioceptive training have shown an average improvement of 52% across motor performance outcome measures (34). This sort of neuromuscular re-education is akin to diaphragmatic training and respiratory muscle strengthening.

In addition, holding poses at full inspiration (e.g., *Bhujangasana or Setu Bandhasana*) imposes an isometric load on the respiratory muscles, effectively acting like inspiratory muscle training. Research on respiratory responses to sustained isometric muscle contraction demonstrated significant increase in FVC and FEV1 following isometric training (35). Research also demonstrated that greater respiratory muscle strength is significantly associated with higher FVC, FEV_1_, and FEF_50_ values, and our *yoga* regimen likely had a similar effect on diaphragm and intercostal strength (36, 37).

Additionally, slow deep audible breathing with glottic constriction (*Ujjayi*) increase end-expiratory lung volume and recruit alveoli in the lung bases through “recruitment maneuvers” that preferentially ventilate lower, gravity-dependent lung zones (38, 39). The physiological mechanisms underlying recruitment maneuvers are well-established: deep breathing patterns re-expand collapsed lung units, restoring their contribution to gas exchange (40, 41). Specific research in post-cardiac surgery patients demonstrated that inspiratory and end-expiratory recruitment maneuvers significantly improved dorsal lung aeration with increase in functional lung volume (42, 43). Forceful exhalations during high frequency breathing (*Kapalbhati*), train the abdominal and internal intercostal muscles; research on diaphragmatic breathing showed significant improvements in PEFR, providing a clear mechanism for the large improvements in expiratory-force-dependent parameters in our study (44). Also, practices like humming (*Bhramari*) have been shown to boost nasal nitric oxide, which acts as a potent endogenous bronchodilator, relaxing airway smooth muscle and improving airway patency (45). The vibratory nature of *Bhramari* and airway resistance of *Ujjayi* stimulate the vagus nerve, which can shift autonomic tone toward parasympathetic dominance through documented changes during *pranayama* practice (46–49). Together, these mechanical, neuromuscular, and autonomic effects form a plausible “cascade” by which *yoga* possibly expanded lung volume, recruited previously under-ventilated regions, and improved airflow, consistent with the large rise in FVC and lung-age reduction.

Furthermore, this study has several limitations. Firstly, as an uncontrolled pre–post design without a comparison group, we could not rule out non-specific effects (e.g. placebo or Hawthorne effects) as contributors to the observed trend. Secondly, the intervention period was fairly short (and follow-up limited), so the durability of these improvements over time is unclear. Finally, outcomes were based solely on spirometry measures; including patient-centered endpoints like dyspnea, exercise capacity, biomarkers or quality of life in future trials would provide a more complete picture. It must be acknowledged that spirometry is a measure of volitional respiratory mechanics and does not directly quantify central respiratory drive (e.g., CO2 rebreathing response or P0.1 occlusion pressure). While our results demonstrate a robust improvement in the mechanical properties of the lung (FVC) and the strength of the respiratory musculature (PEFR), we cannot definitively conclude that the sensitivity of the brainstem chemoreceptors was altered. These limitations suggest that our findings should be interpreted with caution and need confirmation in larger, randomized controlled trials.

## 5 Future directions

Future research should build on these preliminary findings through more rigorous study designs. Controlled or randomized trials, with appropriate comparison arms, are necessary to confirm causal effects and isolate the active components of the *yoga* intervention. Larger, more diverse samples that include women, will be essential for improving generalizability and identifying potential sex-specific or age-related differences in physiological response. Extending follow-up periods is also important to determine the durability of lung-function gains and to assess whether continued practice is required to sustain the benefits. Incorporating objective adherence monitoring and controlling for confounders such as smoking, environmental exposures, or concurrent treatments would strengthen causal interpretation. Additionally, future studies should expand outcome measures beyond spirometry to include markers of respiratory muscle strength, autonomic function, inflammatory biomarkers, exercise tolerance, and clinically meaningful endpoints such as symptom burden or quality of life. Mechanistic studies, using imaging, respiratory muscle electromyography, or measures of ventilatory control (plethysmography or hypercapnic challenge tests), could further clarify how *yoga* reverses opioid-associated restrictive physiology. Together, such advances would provide a more comprehensive understanding of *yoga*’s therapeutic potential for respiratory dysfunction in substance-using populations.

## 6 Conclusion

In this single-arm, pre–post feasibility study, a structured month-long breath-based *yoga* programme produced substantial improvements in lung volumes (notably FVC), expiratory flow (PEFR and mid-flows), and a clinically meaningful reduction in estimated lung age among men with chronic opium dependence. The pattern of results, larger benefits in volume and flow with no change in the FEV_1_/FVC ratio, suggests improved respiratory mechanics and recruitment rather than altered airway obstruction. Given the study’s uncontrolled design, small sample and male-only cohort, these findings should be considered preliminary. However, the intervention’s safety, cultural acceptability, and signal of benefit justify progression to adequately powered, randomized controlled trials that include women, longer follow-up, objective adherence measures, patient-centred outcomes, and mechanistic assessments to establish causality and clinical utility.

## Data Availability

The original contributions presented in the study are included in the article/supplementary material. Further inquiries can be directed to the corresponding author.
